# Targeting excessive avoidance behavior to reduce anxiety related to asthma: A feasibility study of an exposure-based treatment delivered online

**DOI:** 10.1016/j.invent.2021.100415

**Published:** 2021-06-17

**Authors:** Marianne Bonnert, Josefin Särnholm, Erik Andersson, Sten-Erik Bergström, Maria Lalouni, Cecilia Lundholm, Eva Serlachius, Catarina Almqvist

**Affiliations:** a Department of Medical Epidemiology and Biostatistics, Karolinska Institutet, Sweden, Nobels väg 12, 171 77 Stockholm, Sweden; b Centre for Psychiatry Research, Department of Clinical Neuroscience, Karolinska Institutet, Sweden, Norra Stationsgatan 69, 113 64 Stockholm, Sweden; c Department of Clinical Neuroscience, Division of Psychology, Karolinska Institutet, Sweden, Nobels väg 9, 171 65 Stockholm, Sweden; d Pediatric Allergy and Pulmonology Unit at Astrid Lindgren Children's Hospital, Karolinska University Hospital, Stockholm, Sweden; e Department of Clinical Neuroscience, Division of Neuro, Karolinska Institutet, Sweden, Nobels väg 9, 171 65 Stockholm, Sweden; f Stockholm Health Care Services, Region Stockholm, CAP Research Centre, Gävlegatan 22, SE-113 30 Stockholm, Sweden

**Keywords:** Asthma, Anxiety, Avoidance behavior, Cognitive behavior therapy

## Abstract

There is an established relationship between anxiety and asthma, which is associated with poor health outcomes. Most previous cognitive behavior therapies (CBT) have focused on comorbid panic disorder whereas anxiety related to asthma may rather be illness-specific. The feasibility of an online CBT targeting avoidance behavior in anxiety related to asthma was evaluated, using a pretest-posttest design. Thirty participants with self-reported anxiety related to asthma were offered an eight-week treatment with therapist support. Mean adherence was good (80% of content), and most participants (89%) reported adequate relief after treatment. Catastrophizing about asthma (CAS), assessed at 2 months after treatment, improved significantly with a large effect size (Cohen's *d* = 1.52). All secondary outcomes, including asthma control, avoidance behavior, fear of asthma symptoms and quality of life, improved significantly with moderate to large effect sizes (*d*: 0.40–1.44). All improvements were stable at 4 months follow up. Weekly ratings showed that a decrease in avoidance behavior predicted a decrease in CAS the following week throughout the treatment period. We conclude that CBT targeting avoidance behavior is a feasible treatment for anxiety related to asthma. The results justify investigation of efficacy and mechanisms of change in a randomized controlled trial.

ClinicalTrials.gov, ID: NCT03486756.

## Highlights

• Asthma is highly prevalent worldwide and commonly associated with anxiety.
• There is uncertainty as to how best treat anxiety related to asthma.
• Online-CBT for anxiety related to asthma symptoms is acceptable and feasible
• Exposure-based CBT may provide large improvements for anxiety related to asthma.
• Targeting excessive avoidance behavior in anxiety related to asthma seems to be important to reduce anxiety in asthma.

## 1 Introduction

Asthma affects approximately 339 million people worldwide and is globally one of the top 20 causes of disability (Global Asthma Network, 2018). Asthma is characterized by inflammatory processes in the airways leading to structural changes, narrowing of the airways and increased mucus production (McCracken et al., 2017). The changes in the airways cause difficulties to breathe and symptoms such as wheezing, dyspnea and tightness of the chest (Reddel et al., 2014). Many individuals with asthma experience symptoms of anxiety when their breathing is strained, and the association between asthma and anxiety is well established in a number of studies (Brew et al., 2018; Tedner et al., 2016). A diagnosis of asthma and a comorbid anxiety disorder increase the risk for poor asthma control, impaired quality of life as well as increased health care utilization (Braido et al., 2016; Strine et al., 2008). Experimental studies suggest that already subclinical levels of anxiety can affect the perception of asthma. Individuals who have both asthma and elevated anxiety levels have been observed to have trouble distinguishing between them, because the symptoms overlap (Boudreau et al., 2015). Other experimental studies have shown that respiratory symptoms can be triggered due to conditional learning, and that individuals with elevated anxiety levels may have a particular tendency for this type of associative learning (Stoeckel et al., 2015, Stoeckel et al., 2016). Hence, associative learning may cause anxious individuals with asthma to overreact to harmless stimuli. Furthermore, asthma patients are commonly encouraged to avoid triggers for asthma, however the symptom overlap between asthma and anxiety may cause excessive avoidance, leading to impaired daily functioning (Haselkorn et al., 2010), and for some even to work disability and unemployment (Karvala et al., 2018).

Cognitive behavioral therapy (CBT) for asthma and anxiety disorders has shown mixed results (Kew, Kew et al., 2016; Pateraki and Morris, 2018). Most trials have specifically studied asthma patients with a comorbid panic disorder (Feldman et al., 2016; Lehrer et al., 2008; Parry et al., 2012; Ross et al., 2005) using an established panic disorder protocol (Barlow & Barlow and Craske, 2000). The protocol has been modified for the asthma population with an emphasis on relaxation as well as removal of all exercises that could trigger asthma symptoms. Some success on panic disorder symptoms (Ross et al., 2005) and medical adherence (Feldman et al., 2016) was observed. Others have investigated mindfulness therapy for individuals with asthma (Pbert et al., 2012) and relaxation therapy for individuals with increased anxiety (Yorke et al., 2017) with no additive effect compared to control. In summary, even though there are some promising results, further treatment development and refinement warranted.

Previous CBT may be based on the early findings that panic disorder is a risk factor for a worsened asthma prognosis (Dahlem et al., 1977; Dirks et al., 1977), and that this may be due to an illness-specific panic fear that increases attention to asthmatic symptoms (Kinsman et al., 1980). These studies were followed by an increased focus on the interaction between panic disorder and asthma (Feldman et al., 2009). However, recent experimental studies have demonstrated that sub-clinical anxiety might be enough to complicate the perception of asthma symptoms (De Peuter et al., 2005; Janssens et al., 2015). Thus, anxiety in asthma may not be related to one established anxiety disorder, such as panic disorder, but could be affected by a propensity to react with anxiety toward respiratory symptoms. If so, the treatment would need to be broadened to include individuals with sub-clinical levels of anxiety specifically related to asthma.

The gold standard intervention to treat anxiety is exposure-based CBT (Hofmann et al., 2012). In this treatment, a behavioral pattern of avoidance is targeted and reduced in order to overcome excessive fear of anxiety-provoking stimuli and situations (Craske et al., 2014). Exposure-based CBT is effective for several somatic disorders where fear of symptoms may lead to undesirable avoidance (Hedman-Lagerlöf et al., 2018; Lalouni et al., 2019; Ljótsson et al., 2011; Särnholm et al., 2017). Consequently, there is reason to investigate whether exposure-based CBT can be feasible and potentially effective for anxiety related to asthma. However, exposure treatment of asthma is complicated by the fact that asthma-triggers should be avoided, and medical adherence is important to maintain asthma control. It is therefore of utmost importance to specifically design an exposure treatment for anxiety related to asthma.

In a pre-study (*N* = 6), we have previously developed a treatment for anxiety related to asthma based on individual behavioral analyzes (Bonnert et al., 2020). We observed a pattern of increased reactivity to respiratory symptoms and behavioral avoidance of any asthma-like symptoms. Hence, we developed a treatment based on exposure exercises targeting excessive avoidance and fear of asthma-like symptoms (e.g., encouraging physical exercise to expose for asthma-like symptoms such as increased breathing). In the current study, the treatment was standardized for online delivery to increase accessibility for a disorder few psychologists are trained to treat. The objective was to assess the feasibility and potential efficacy of the online-CBT. Another aim was to explore if the target in treatment, a reduction in excessive avoidance behavior elicited by asthma anxiety, would precede a later reduction in anxiety related to asthma.

## 2 Methods

### 2.1 Design

This was a feasibility study with a pretest-posttest design (*N* = 30). All participants were offered online-CBT. All assessments were conducted online without the influence of study staff. The platform used for treatment and online assessments was specifically designed for the purpose. Participants received automatic text-message reminders to login at the time for each assessment. All participants received unique personal logins to ensure integrity, and double authentication with a temporary password was used at each login. Ethical approval by the Regional Ethical Review Board in Stockholm was received in March 2018.

### 2.2 Eligibility criteria

Eligible participants had: (a) a physician's diagnosis of asthma, (b) reported worry about asthma, i.e., fear of asthma symptoms that elicited excessive avoidance behavior, (c) age 18–75, (d) no severe somatic disorder or comorbid respiratory disorder, (e) no severe psychiatric disorder, (f) no hazardous use of drugs or alcohol, (g) no on-going psychological treatment, (h) no or stable psychotropic medicine, (i) internet-access and access to computer or a smart-phone.

### 2.3 Procedure

Participants were recruited through advertisements in Stockholm from April to October 2018. Participants who completed online screening were invited to a clinical interview over the telephone with a psychologist, after which informed consent was submitted by mail. During the interview, eligibility criteria were examined and psychiatric comorbidity was assessed with the Mini-International Neuropsychiatric Interview (M.I.N.I.; Sheehan et al., 1998).

#### 2.3.1 Data collection

Data was collected pretreatment, weekly, posttreatment, 2 months after treatment completion (primary endpoint) and 4 months after treatment completion. See Supplement 1 for a detailed overview of all assessments.

#### 2.3.2 Assessments during the inclusion procedure

Before inclusion in the study, participants answered the following instruments in the on-line screening: The Alcohol Use Disorder Identification Test (Saunders, Aasland, & Babor, 1993) and the Drug Use Disorder Identification Test (Berman, Bergman, Palmstierna, & Schlyter, 2003) to identify problematic alcohol or drug use. In addition, participants answered questions about the severity and diagnosis of asthma, allergies, asthma medication, and demographic information. For an initial identification of participants with anxiety related to asthma eligible participants were required to answer one of the following statements in the affirmative: “I'm worried about my asthma” (yes/no). “I feel hindered in my daily life because of my asthma” (yes/no). During the clinical interview, the psychologist further examined whether it was a matter of excessive fear and avoidance of asthma symptoms. Participants who had no such fear were not considered to have anxiety related to asthma and were excluded from the study.

### 2.4 Feasibility measures

*The Credibility Rating Scale* (C-scale; Borkovec and Nau, 1972) assessed treatment credibility at week 2. The C-scale comprise five items (e.g., “How logical does this type of treatment seem to you?” and “How confident would you be to recommend this treatment to a friend with similar problems as yours?”) on an 11-point scale from 0 (*not at all*) to 10 (*very*). *The Working Alliance Inventory* (WAI; Horvath & Greenberg, 1989) assessed perceived alliance at week three. For this study we used the six-item version on a seven-point scale from 1 (*never*) to 7 (*always*), adapted for Online-CBT (Falkenström, Hatcher, Skjulsvik, Larsson, & Holmqvist, 2015), including items such as “I feel that my therapist appreciates me” and “My therapist and I are working toward mutually agreed upon goals.” Treatment satisfaction was assessed at week eight with the *Client Satisfaction Questionnaire* (CSQ-8; Attkisson & Attkisson and Zwick, 1982). The CSQ-8 is rated on a four-point scale from 0 (*dissatisfied*) to 3 (*satisfied*). Adequate relief was assessed at week eight with the *Subjective Assessment Questionnaire* (SAQ; Gonsalkorale, Gonsalkorale et al., 2003). SAQ comprise one item about overall symptom severity posttreatment compared to pretreatment rated from 0 (*much worse*) to 6 (*much better*). *Adverse events* (*AE*) were assessed after treatment completion. Participants described unwanted effects during the treatment period and rated impact from 0 (*no negative effect at all*) to 3 (*very negative effect*) when the event occurred as well as lingering impact at the time for the assessment.

### 2.5 Measures for clinical effect in the sample

#### 2.5.1 Primary outcome

*Catastrophizing about asthma* (CAS; De Peuter et al., 2006) comprise 24 statements assessing catastrophizing thoughts during an asthma attack and in daily life rated from 0 (*not at all*) to 4 (*certainly*). Examples of items are “During an asthma attack I worry all the time whether the asthma attack will end” and “In general, when I do not have an attack, I keep thinking about how bad my asthma is”. The CAS was validated in a clinical sample and demonstrated good psychometric properties with a high internal consistency (Cronbach's alpha = 0.93) and excellent test-retest reliability (*r* = 0.94), as well as a moderate correlation (*r* = 0.30) with perceptions of asthma symptoms The CAS was used to assess anxiety specifically related to asthma. The English version was translated to Swedish by two of the authors prior to the study.

#### 2.5.2 Secondary outcomes

The *Asthma Control Test* (*ACT*; Nathan et al., 2004) uses five items on a five-point scale to assess asthma control. It is an often used scale with good psychometric properties (Schatz et al., 2006). The following cut-offs were used; ≤15 p = uncontrolled asthma; 16–19 p = partially controlled asthma; ≥20 p = controlled asthma (Schatz et al., 2006). *Fear of asthma symptoms* (*FAS*) is a scale under validation developed by the research group and includes items such as “Whenever I feel symptoms of asthma, I become anxious” and “I'm always aware of my breathing.” FAS consists of 13 items on a scale from 0 (*Do not agree at all*) to 5 (*Very much agree*). The internal consistency in the sample was high (Cronbach's alpha = 0.83). The *Asthma behavior checklist* (*ABC*) is developed to assess asthma related behavioral avoidance and consists of items such as: “(*Due to my asthma*) I often check my breathing”, “(*Due to my asthma*) I avoid taking walks with other people”. The ABC is under validation and comprise 31 statements on a 7-graded scale, from 0 (never) to 7 (always). In this sample, we saw high internal consistency (Cronbach's alpha = 0.90). *The Penn State Worry Questionnaire* (PSWQ; Meyer, Miller, Metzger, & Borkovec, 1990) is a well-used and validated instrument that comprise 16 questions about worry. *Anxiety Sensitivity Index*-*3* (ASI; Wheaton et al., 2012) is an 18-item scale, rated between very little (0 points) to very much (4 points). The ASI has previously been used to measure anxiety-sensitivity in CBT for asthma (Kew et al., 2016). The Perceived Stress Scale (PSS-10; Cohen, 1988) is a well-used ten-item scale assessing perceived distress in daily life, with demonstrated good psychometric properties (Lee, 2012). Quality of life was assessed with the validated scale *Brunnsviken Brief Quality of Life Scale* (BBQ; Lindner et al., 2016). The BBQ has 12 items about six important life areas, and ratings between 0 (*Do not agree at all*) to 6 (*Agree completely*).

#### 2.5.3 Objective measure of lung function

Asthma Tuner®, was added to assess lung function (Forced expiratory volume, FEV1) at pretreatment compared to posttreatment. The digital spirometer is a small device that is connected to a smartphone application. Asthma Tuner has been evaluated in a primary care population (Ljungberg et al., 2019). The device was posted to the participants with instructions to use the Asthma Tuner daily for five days before pretreatment and five days after posttreatment.

### 2.6 Treatment

The 8-week online treatment was therapist-guided with at least weekly short written messages from therapists. Therapists were two psychologists (MB & JS) with extensive experience of Internet-CBT for somatic disorders. The participants worked independently with the modules and planned their own homework. Therapist messages aimed to encourage individual exercises and to answer participants' questions about the treatment content. The therapists reviewed the participants' work with their individual exposure exercises online and made suggestions through written text-messages if there were signs of deterioration of symptoms. The therapist answered participants' messages within 48 h. All content and messages were administered online.

#### 2.6.1 Treatment content

**Fig. 1:**
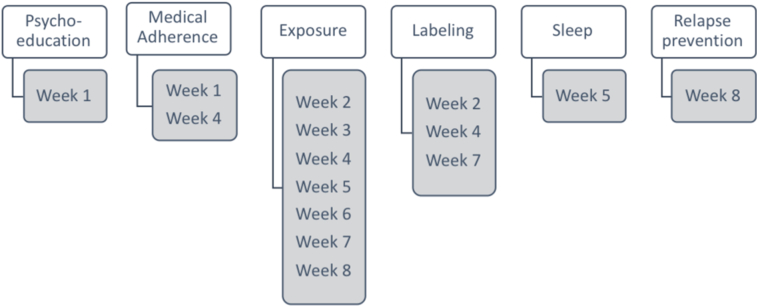
Treatment components.

**Fig. 2:**
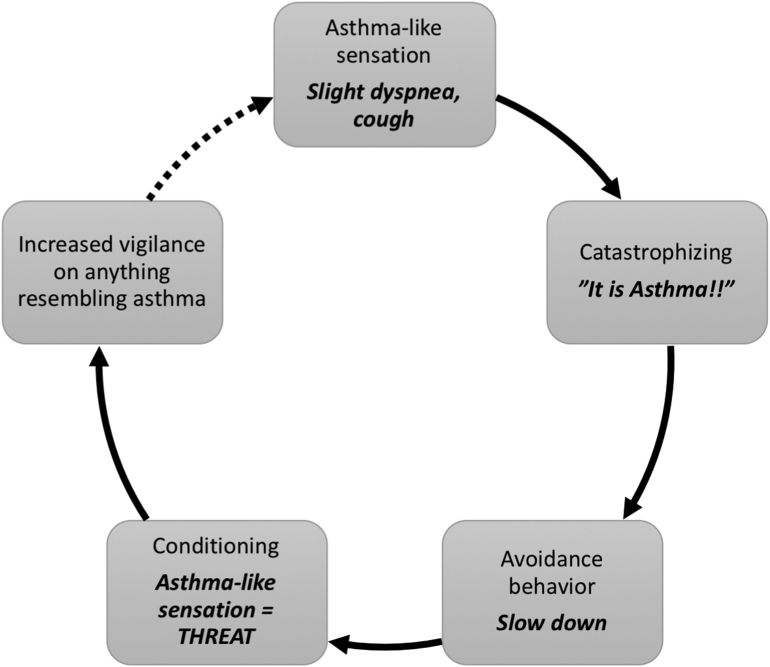
Explanatory model.

The treatment comprised weekly modules, see Fig. 1 for overview. In the first module, education about asthma was presented and adherence to prescribed asthma medication and action plans was emphasized. Subsequently, an explanatory model was presented in which excessive fear of asthma and subsequent avoidance of asthma-like symptoms is described as maintaining factors, see Fig. 2. In the second module, participants mapped avoidance behaviors, were taught affective labeling (i.e., awareness of current asthma-like symptoms, thoughts, feelings and behavioral impulses) to reduce immediate negative reactivity. The second module included a behavioral experiment (e.g., taking the stairs instead of the elevator) to gather information for behavioral change. The third module consisted of the principles of exposure and examples of how to challenge fear of asthma-like symptoms through exposure exercises. Participants could for example expose for the fear of talking in front of others in the presence of asthma-symptoms, or take the stairs and risk increased breathing, or take the bus and risk coughing (this was before the Covid-19 pandemic outbreak). From the third to the seventh module participants were encouraged to perform regular exposure exercises to avoided situations and activities. In planning the exposure exercises the participants had the help of clinical vignettes and examples and an individualized weekly plan for exposure exercises. The last module consisted of a summary of the treatment, and relapse prevention.

### 2.7 Data analysis

Multilevel linear mixed models (MLMM) were performed in STATA 15.1 to test the effects of time from baseline to the primary endpoint at 2 months follow-up. The analyses of weekly assessments included ten time-points: baseline (week 0), week 1–7, posttreatment (week 8), and 2 months follow-up. Cohen's *d* effect sizes with 95% confidence intervals were calculated using the mean difference between the observed means at each endpoint divided by the pooled standard deviation. Clinically significant change was defined as ≥50% improvement on the primary outcome, and a significantly larger proportion of controlled asthma at pretreatment compared to the primary endpoint investigated by Chi2. To investigate the relationship between the target in treatment, avoidance behavior (ABC), and the primary outcome (CAS), weekly measures (baseline-week8) were used in MLMM analyses. The hypothesis was that a change in ABC would predict change in CAS the subsequent week, but not the other way around.

#### 2.7.1 Power

We aimed to include 30 participants to be able to detect at least a moderate within group effect size (Cohen's *d* ≥ 0.50), with a power of 0.80 and alpha-level 0.05.

## 3 Results

### 3.1 Inclusion, adherence and attrition

**Fig. 3:**
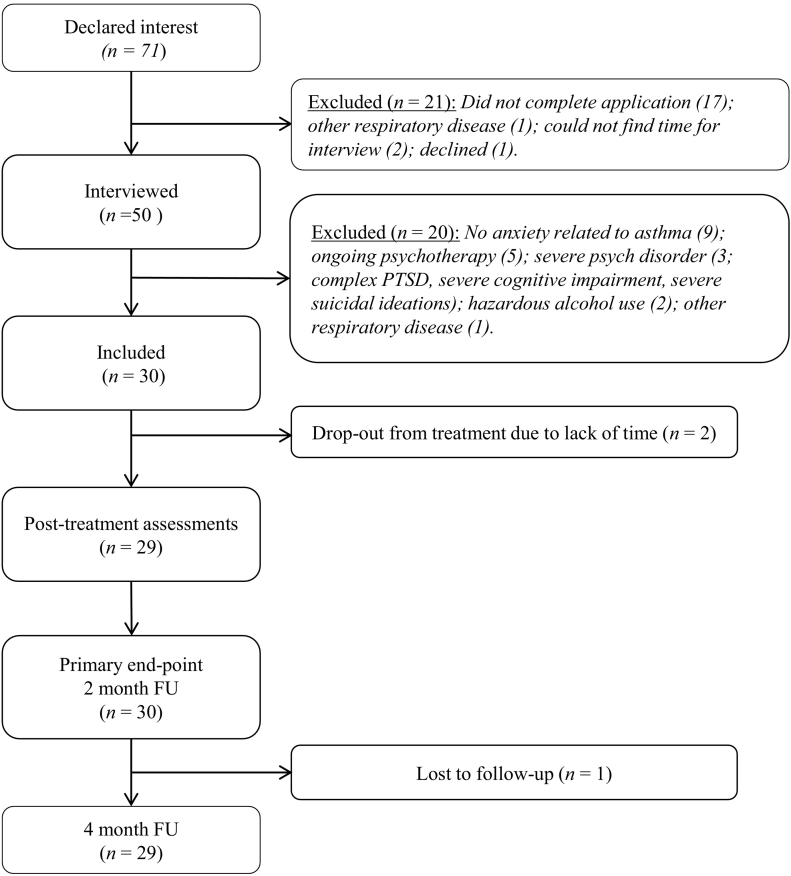
Study flow chart.

After completing screening, fifty individuals were interviewed of which thirty were included in the study (Fig. 3). As seen in Table 1, most participants were female (83%), and mean age was 52 years (range: 21–74). Before treatment, two participants withdrew due to time restraints. Both agreed to answer all assessments and were included in the analyses using the principle of intent to treat (ITT). Participants completed on average 6.4 out of 8 modules (80.4%). The mean therapist time was 47 min (SD = 25.76) per participant or 7.23 min (SD = 3.04) per module.

**Table 1:** Baseline characteristics (*N* = 30).

Age, mean (SD), range	52 (15,08), 21–74 years
Female, n (%)	26 (83%)
Education	
High school, n (%)	13 (43%)
University, n (%)	16 (53%)
Other, n (%)	1 (4%)
Asthma	
Duration, mean (SD), range	18.48 (16.80), 1–48 years
Controlled asthma (ACT ≥20), n (%)	4 (13%)
Partially controlled asthma (ACT 16–19), n (%)	8 (27%)
Uncontrolled asthma (ACT ≤15), n (%)	18 (60%)
Asthma medication	
SABA^a^	19 (63%)
ICS^b^	11 (37%)
ICS and LABA^c^	18 (60%)
LTRA^d^	5 (17%)
>1 category of meds., n (%)	20 (67%)
Allergy	26 (83%)
Pollen allergy (e.g., hay fever), n (%)	19 (63%)
Allergy to furred pets, n (%)	11 (37%)
Eczema, n (%)	7 (23%)
Food allergy, n (%)	8 (27%)
Other, n (%)	13 (43%)
Psychiatric disorders M.I.N.I, n (%)	22 (73%)
>1 diagnosis M.I.N.I., n (%)	15 (50%)
Depression, n (%)	2 (0,7%)
Panic disorder, n (%)	9 (30%)
Agoraphobia, n (%)	9 (30%)
Social anxiety disorder, n (%)	7 (23%)
OCD, n (%)	2 (0,7%)
GAD, n (%)	13 (43%)
Psychotropic medicine^e^, n (%)	5 (17%)

a Short-acting beta agonists.

b Inhaled corticosteroids.

c Long-acting beta agonists.

d Leukotriene receptor antagonists.

e Pain analgesics, Mirtazapin and SSRI.

### 3.2 Feasibility outcomes

Participants found the treatment credible (C-scale, mean = 35 out of 50; SD = 7.43). Perceived working alliance with the online therapist was in the higher range (WAI, mean = 33 out of 42; SD = 8.34). Satisfaction with the treatment (CSQ; Mean = 20 out of 24; SD = 3.06) and subjective adequate relief (SAQ) was high (Mean = 5 out of 6; SD = 0.97). None reported worsened symptoms. Four (14%) participants reported adverse events connected to treatment, However, the negative effects were short-term and of harmless nature (such as increased irritability, increased awareness of breathing) and overall rated as mild.

### 3.3 Clinical effect

There was a significant decrease [*β* = −1.37 (s.e. = 0.116), *p* < .001] with a large effect size (Cohen's *d* = 1.52 [CI: 0.94, 2.09]) in the primary outcome, catastrophizing about asthma, from pretreatment to the primary endpoint at 2 months after treatment completion. See Table 2 for full results. The improvement was stable four months after treatment.

**Table 2:** Detailed results from the multilevel linear mixed models analyses with beta-values, standard errors, *p*-values, estimated means, standard deviations, and Cohens'd effect sizes.

Measure	Pre (*n* = 30) Mean (SD)	Post (*n* = 29) Mean (SD)	2 mo FU (n = 30) Mean (SD)	Beta (s.e.), p-value	Pretreatment – 2 mo FU Cohen's *d* [CI]	4 mo FU (n = 29) Mean (SD)
Catastrophizing about asthma (CAS)	37.06 (14.49)	20.62 (14.04)	15.46 (13.87)	−1.37 (0.116), 0.000	1.52 [0.94, 2.09]	15.62 (13.71)
Asthma control (ACT)^a^	15.0 (3.94)	19.03 (3.94)	19.97 (2.88)	0.28 (0.042), 0.000	1.27 [0.71, 1.82]	20.28 (3.66)
Fear of asthma symptoms (FAS)	28.6 (11.14)	15.17 (9.64)	12.79 (10.76)	−1.14 (0.101), 0.000	1.44 [0.86, 2.01]	14.48 (10.56)
Avoidance behavior (ABC)	95.3 (28.25)	74.70 (26.85)	69.10 (30.18)	−2.02 (0.201), 0.000	0.90 [0.36, 1.42]	70.58 (26.98)
Percieved stress (PSS-10)	19.17 (6.46)	13.18 (8.46)	14.43 (7.55)	−0.30 (0.093), 0.002	0.67 [0.15, 1.91]	14.97 (9.09)
Generalized worry (PSWQ)	54.37 (9.73)	48.17 (11.17)	43.68 (10.82)	−0.67 (0.088), 0.000	1.04 [0.49, 1.59]	46.21 (11.01)
Anxiety sensitivity (ASI)	22.87 (11.02)	16.46 (9.93)	14.33 (8.86)	−0.53 (0.119), 0.000	0.85 [0.32, 1.38]	14.41 (8.81)
Quality of life (BBQ)^a^	60.23 (18.32)	69.07 (18.61)	67.76 (18.96)	49 (0.157), 0.002	0.40 [−0.11, 0.92]	73.07 (21.52)

Cohens d is the standardized mean difference, d ≥ 0.2 corresponds to a small effects size, d ≥ 0.5 corresponds to a moderate effect size and d ≥ 0.8 corresponds to a large effect size.

a Improvements yield increased values.

#### 3.3.1 Secondary outcomes

There were significant improvements with large effect sizes from pretreatment to the primary endpoint in asthma control (*d* = 1.27), fear of asthma symptoms (*d* = 1.44), avoidance behavior (*d* = 0.90), generalized worry (*d* = 1.04) and anxiety sensitivity (*d* = 0.85). Perceived stress decreased with a moderate effect size (*d* = 0.67). All improvements were stable at four months. Quality of life improved significantly with a small effect size at the primary endpoint (*d* = 0.40) and was further improved 4 months after treatment (*d* = 0.64).

#### 3.3.2 Clinically significant change

At the primary endpoint 21 participants (70%) had at least a 50% reduction in the primary outcome. Furthermore, the proportion of participants with controlled asthma (ACT≥20) was 65% (*N* = 19) at the primary endpoint compared to 19% (*N* = 4) before treatment (*X*^*2*^ = 17.0, p < .001).

### 3.4 Objective measure of lung-function

Twenty-five participants connected the app to the digital spirometer Asthma Tuner and contributed with data from 2 to 5 days at baseline (Mean = 4.5). The remaining five participants had difficulties to install the app. At posttreatment 18 participants contributed with data (range 1–5 days, Mean = 2.2). The mean FEV1 at pretreatment was 2.53 (SD = 0.75) and the mean FEV1 at posttreatment was 2.61 (SD = 0.58), a non-significant difference (*p* = .754).

### 3.5 Relationship avoidance and catastrophizing

Multilevel mixed model analyses showed that avoidance behavior at a given week predicted degree of catastrophizing the following week (*ß* = 0.89, z = 24.33, *p* ≤ .001). However, catastrophizing did not predict change in avoidance (*ß* = −0.01, z = −0.21, *p* = .83).

## 4 Discussion

In this study we investigated feasibility and potential clinical efficacy of an online-CBT for anxiety related to asthma targeting avoidance behavior. Participants found the treatment helpful and satisfying. Adherence was acceptable and data attrition was very low. Analysis of clinical efficacy in the sample demonstrated significant improvements in all outcomes, with large effect sizes in catastrophizing about asthma and the outcomes measuring symptom fear, avoidance behavior, generalized worry and anxiety sensitivity. The improvements seemed to be of clinical significance, there was ≥50% reduction in catastrophizing about asthma in more than two thirds of the participants, and a significantly larger proportion of participants with controlled asthma after treatment than before. Improvements were stable or further improved at follow-up. Hence, online-CBT for anxiety related to asthma is feasible and has the potential to largely reduce catastrophizing about asthma, increase asthma control, reduce anxiety and avoidance behavior, as well as to improve quality of life. The investigation of the role of avoidance behavior in this sample lends preliminary support for the treatment model, targeting avoidance to reduce anxiety related to asthma.

The early findings of Dirks and Kinsman on illness-specific panic fear in asthma were followed by a focus on CBT for panic disorders comorbid with asthma (Feldman et al., 2009). Hence, most previous studies on CBT for asthma and anxiety have only included participants with a clinical diagnosis of panic disorder (Feldman et al., 2016; Pbert et al., 2012; Put et al., 2003; Ross et al., 2005). In the current study, we included all who reported worry about asthma, based on experimental research indicating that sub-clinical anxiety may be sufficient for over-perception of asthma symptoms. In this study, we saw multiple anxiety disorders within the group. A similar spread of anxiety disorders in asthma has been reported previously (Nascimento et al., 2002). Our results support a broader inclusion to reach all with anxiety specifically related to asthma.

The exposure-based treatment can work through several mechanisms; the tolerance of internal sensations (Alsaid-Habia et al., 2019) may increase as a result of repeated systematic exposure and the ability to differentiate between asthma and anxiety symptoms (Janssens et al., 2009) could improve through labeling that decreases negative reactivity. Improved differentiation may lead to increased asthma control as fewer symptoms are interpreted as asthma. Importantly, because associative learning can be heightened in anxiety-related asthma (De Peuter et al., 2005), the exposure exercises might lead to symptomatic improvement through a reduction in avoidance behavior, eliciting an inhibitory response that may decrease the threat potentiation of asthma-like symptoms (Sucec et al., 2019). In the current study avoidance behavior uni-directionally predicted level of catastrophizing the coming week. Reduced avoidance behavior is a mediator in exposure-based CBT for pain (Bonnert et al., 2018; Hedman-Lagerlöf et al., 2019). Reducing excessive avoidance behavior for asthma may therefore also be important for individuals with anxiety related to asthma in order to improve the perception of asthma-symptoms. A reduction in fear of asthma symptoms could thus be caused by repeatedly exercising calm and adequate behavior in the presence of asthma-like symptoms. The mechanisms need to be further investigated in a properly designed mediational trial.

We couldn't detect any change in lung function with the objective digital spirometer Asthma Tuner. Even though this result is somewhat hampered by data loss due to installation difficulties, it is an indication that the treatment, encouraging participants to expose for asthma-like symptoms, is safe. The unaltered lung function in this study is in line with Pbert et al. (2012) who saw no significant change after CBT. An objective measure of lung function is an important feature as a security measure when investigating new treatments, even though change may not be expected.

A few participants did report some mild adverse events due to treatment. The exercises in exposure-based CBT can be challenging for some, as participants are encouraged to change a habitual pattern of behavior, which may initially increase anxiety and other sensations that many have long struggled to avoid (Himle et al., 2003). This pattern may be reflected in the type of reported events. The fact that almost all participants were satisfied after completion of treatment strengthens the acceptance and feasibility of the treatment.

The design is an obvious limitation in this study; without a control condition we cannot rule out that the observed improvements are due to a decrease in asthma symptoms apart from the treatment. Nonetheless, the choice of design was reasonable given the novelty in treatment approach and the aim to investigate feasibility. Furthermore, since medical adherence was emphasized at the beginning of treatment, this may have affected the outcome. In future studies, the potential effect of information about asthma and medical adherence should be controlled. Even though we saw large improvements in catastrophizing about asthma, the results need to be interpreted with caution as no validation study has yet been performed on sensitivity to change on the CAS. However, all other scales used show similar improvements, which may support the overall results found in our study. The participants did get feedback on FEV1 when using the Asthma Tuner, which may have caused some to use the Asthma Tuner as a security behavior. However, FEV1 did not change at all from pretreatment to posttreatment, while all other scales demonstrated improvement. Thus, there are no obvious signs of a relationship. Any potential effect that the Asthma Tuner may have on anxiety related to asthma would need further investigation in a randomized controlled trial. Seasonal change in asthma severity is normal due to pollen and seasonal flu. However, the six-month continuous recruitment distributed assessments over seasons, which controlled for any temporary seasonal impact. The very low data attrition increases the precision of the results and is a clear strength of this study.

## 5 Conclusions

Online-CBT targeting excessive avoidance behavior for anxiety related to asthma is an acceptable, feasible and safe treatment that may considerably improve catastrophizing about asthma, asthma control, fear of asthma symptoms, avoidance behavior, anxiety sensitivity, generalized worry and also reduce stress and improve quality of life. Targeting excessive avoidance behavior in anxiety related to asthma seems to be important to reduce anxiety in asthma. To ensure efficacy, the results need to be confirmed in a randomized controlled trial.

The following is the supplementary data related to this article.

Supplement 1 Timetable over assessments of feasibility and potential efficacy. Supplement 1

## Declaration of competing interest

The authors declare that they have no known competing financial interests or personal relationships that could have appeared to influence the work reported in this paper.
