# Supplementary material for: Targeting excessive avoidance behavior to reduce anxiety related to asthma: A feasibility study of an exposure-based treatment delivered online
Source: Internet Interv. 2021 Jun 17;25:100415. doi: 10.1016/j.invent.2021.100415 (PMC8350602; doi:10.1016/j.invent.2021.100415)
Supplement: Supplement 1 — Timetable over assessments of feasibility and potential efficacy. [file mmc1.docx]

Supplement 1. Timetable over assessments of feasibility and potential efficacy.

| Time  Outcome | Pre-treatment (week 0) | During treatment  (week 1-7) | Post-treatment  (week 8) | Follow up  2 month  (week 16) | Follow up  4 month  (week 32) |
| --- | --- | --- | --- | --- | --- |
| Feasibility |  |  |  |  |  |
| *Credibility, c-scale* |  | **X** (week 2) |  |  |  |
| *Working alliance, WAi* |  | **X** (week 4) |  |  |  |
| *Satisfaction, csq* |  |  | **X** |  |  |
| *Subjective adequate relief, SAQ* |  |  | **X** |  |  |
| *Adverse events* |  |  | **X** |  |  |
| Potential efficacy |  |  |  |  |  |
| *Catastrophizing about asthma, CAS* | **X** | **X** | **X** | **X** | X |
| *Asthma Control test, ACT* | **X** | **X** | **X** | **X** | X |
| *Fear of asthma symptoms, FAS* | **X** | **X** | **X** | **X** | X |
| *AVoidant behavior, ABC* | **X** | **X** | **X** | **X** | X |
| *Perceived stress, PSS-10* | **X** |  | **X** | **X** | X |
| *Generalized worry, PSWQ* | **X** |  | **X** | **X** | X |
| *Anxiety sensitivity, ASI* | **X** |  | **X** | **X** | X |
| *Quality of life, BBQ* | **X** |  | **X** | **X** | X |
| *FEV1, Asthma-tuner* | X |  | X |  |  |
